# Feasibility and robustness of an oral HIV self-test in a rural community in South-Africa: An observational diagnostic study

**DOI:** 10.1371/journal.pone.0215353

**Published:** 2019-04-15

**Authors:** Walter Devillé, Hugo Tempelman

**Affiliations:** 1 Ndlovu Care Group, Groblersdal, South Africa; 2 Julius Global Health, Julius Center for Health Sciences and Primary Care, University Medical Center Utrecht, Utrecht, The Netherlands; Ghent University, BELGIUM

## Abstract

**Background:**

HIV-self-testing (HIVST) could be a strategy to get more people tested for HIV in resource limited settings. One of the prerequisites of a successful HIVST programme is the availability of an easy to use, valid HIV-test which is robust against field conditions and procedural errors by untrained lay users.

**Methods and findings:**

The primary objective of this study was to evaluate the ability of untrained persons to correctly interpret the OraQuick HIV Self-Test results with oral fluid compared with results obtained by trained users using the matched lot OraQuick Rapid HIV-1/2 Antibody Test and blinded to the results of the Self-Test. Sensitivity of the OraQuick HIV Self-Test in untrained users was 101 in 102 (99.02%; 95%CI = 93.88–99.95%)—and specificity– 1,241 in 1,241 (100.0%; 95%CI = 99.62–100.0%). Forty-eight Self-Tests were excluded in the accuracy analysis (due to a result read as invalid, not sure or ambiguous) resulting in a test system failure rate of 3.45% (95% CI 2.56%-4.55%). At least one observation of difficulty or error with one or more of the test steps were seen in 1,193 (84.6%) participants. Age, education and health literacy were independently associated with the sum score of procedural errors and difficulties. Four tests did not provide a valid result as determined by the trained user’s interpretation of the Self-Test.

**Conclusions:**

The OraQuick HIV Self-Test provides reliable and repeatable results in a rural field environment in spite of procedural errors.

## Introduction

In 2014, UNAIDS launched the global campaign 90-90-90 aiming that at least 90% of people infected by HIV should be aware of their status by 2020 [1]. If the campaign resulted in having 90% of HIV positive persons in treatment and 90% virological suppressed, it would be a step toward preventing the further spread of the epidemic. Globally 70% of HIV infected people are aware of their status [2]. In South-Africa, still experiencing one of the major HIV epidemics, various HIV testing efforts and strategies resulted in 2014, in 48% of the estimated 7 million HIV+ persons being on antiretroviral treatment and 79% of treated patients being virally suppressed [3]. In 2015, more than 10 million people in South-Africa were tested for HIV (19% of the population) [3].

Conventional HIV testing and counselling (HTC) is usually limited to provider initiated testing and counselling (PITC) in health care facilities or voluntary client-initiated HTC by outreach programmes such as home based testing. This is also the case in South-Africa [4]. Participation in the conventional programmes is limited by fear of stigmatization and mistrust in health care providers [5, 6, 7]. HIV self-testing (HST) may be an innovative new strategy to help scale up HIV testing [8]. A systematic review about the acceptability of HIV-self-testing (HST) in resource limited as well as high income countries concluded that it is an acceptable testing strategy and that it can be performed accurately by the majority of the self-testers [9]. Another systematic review concluded that both supervised (self-testing aided by a health care professional) and unsupervised (performed by self-tester) self-testing strategies were highly acceptable and equally preferred [10].

One of the prerequisites for a successful self-testing programme is the availability of an easy to use, valid HIV-test which is robust against field conditions and procedural errors by untrained lay users [11]. The test should be usable by various populations in high income as well as in resource limited countries among populations with different educational backgrounds and health literacy levels. Peck et al. [12] conducted a usability study of 5 test prototypes in unsupervised self-testing in Kenya, Malawi and South-Africa. Common errors identified in this study included errors in sample collection and errors in interpretation of test results. The authors recommended the use of pictorial instructions that are easy to understand, simple sample collection, fewer steps, and results that are easy to interpret [12]. One of the tests was an oral HIV self-test leading to less errors compared to the fingerstick self-test prototypes.

An oral HIV self-test (O-HIVST) distributed in South Africa is the OraQuick ADVANCE HIV-1/2 Rapid Antibody Test (Orasure Technologies Inc) [13]. The Self-Test is now being used in high income countries where it has been shown to have a high sensitivity/specificity when used by untrained persons [10]. It is necessary to investigate the validity of the same Self-Test when used by a rural population with little formal education. Accurate results were obtained with a test run from an oral mucosal transudate specimen by adults in a poor urban setting in Malawi after a demonstration and the use of illustrated instructions (sensitivity of 93.6% (95% CI:88.2%–97.0%) and specificity of 99.9% (95% CI:99.6%–100%)). In the same study procedural errors were identified in 10% of the participants [14]. In 2015, in rural South Africa, supervised OraQuick self-testing showed a sensitivity of 98.7% (95% CI 96.8–99.6), and specificity was 100% (95% CI 99.8–100) [15]. The same study reported high agreement in counsellor and participant reading of O-HIVST results and high participant compliance with self-testing procedures (error rate 0.09%)

Despite the above mentioned results, more evidence on acceptability, feasibility, and accuracy of home self-testing without supervision or active introduction by a counsellor in a rural population in South Africa is necessary to come as close as possible to real life circumstances. [10, 16]. The primary objective of this study was to evaluate the ability of untrained persons to correctly interpret their test results using the OraQuick HIV Self-Test compared with results obtained by trained users using the matched lot OraQuick Rapid HIV-1/2 Antibody Test.

## Materials and methods

### Design

This was a diagnostic evaluation study, evaluating the adequacy of the packaging and labelling to direct sample collection, test performance, and reading and interpretation of oral fluid test results for the OraQuick HIV “Self-Test” by the untrained study participant conducting the Self-Test. The observer was not allowed to give any explanation about the use of the test, except for during instances when the participant was at risk to harm themselves. The observer used a list of questions to record observed difficulties and procedural errors in using the test. The waiting time before the test result was read was also recorded.

The comparator reference test was the study participant’s oral fluid result in the professional use OraQuick Rapid HIV-1/2 Antibody Test read by a counsellor or nurse trained in the use of the test and blinded to both the study participant’s Self-Test result and the observer’s interpretation of the study participant’s Self-Test. The blinded reader was sitting in a separate room.

After being informed about the purpose of the study and having consented to the study and HIV testing, eligible participants were enrolled into the study. Using a questionnaire, demographic data were collected, as well as health literacy, information about health status and medication use, visual impairment, oral food and drink intake or the use of other oral products. Participants were informed that they would receive a package with the oral test with instructions in various languages inside and that they had to test themselves under observation of one of the team members. They were told that after having tested themselves, the observer would take a second oral sample to be compared independently with the first one by another team member.

Any participant who had a preliminary positive result with either the Self-Test or the OraQuick Professional Test, would also have consecutively a finger prick rapid test, as per the HIV testing algorithm in South Africa. In the event that this confirmatory rapid test was HIV positive (Rapid Reactive), the study participant was referred to a clinical site of their choice for further diagnosis, treatment and care. In the event the second rapid test showed a negative result, the HIV status was considered indeterminate, a blood sample was drawn and the study participant was referred to a clinical site for the results of the HIV ELISA test and clinical follow-up.

All participants received post-test counselling and upon completion of the test participants with negative test outcomes were asked about their plans regarding future HIV testing to understand the acceptability of the Self-Test.

### Setting

Communities in the Moutse area, Sekhukhune District, Limpopo Province (South Africa), were informed about the outreach HIV Counselling and Testing programme. In 2012, HIV prevalence in this rural community was between 9 and 14% [17]. The clinical site connected to the Ndlovu Research Center was Ndlovu Medical Clinic in Elandsdoorn, Limpopo province, a well experienced and trusted ART treatment site with over 3,600 HIV patients on treatment. The Ndlovu Medical Clinic has a coverage area of 30 km around the clinic. HIV testing and counselling is provided at the clinic and through outreach events voluntarily and free of charge. Participants for this study were invited through outreach events in the villages and at the mall in the Moutse area or at Health Awareness activities organized by the Department of Health. An outreach team composed of nurses and counsellors went to the communities between February and July 2016.

### Participants

Eligible study participants were those with unknown HIV status in the age group 18 to 49 years and who were able to provide informed consent. Candidates who had tested for HIV within 6 months of enrolment, who were trained users or other study participants such that they would have had familiarity with conducting a rapid oral HIV test, who had received experimental HIV vaccination or were on antiretroviral treatment (ARV) or Pre-exposure Prophylaxis (PreP) were considered ineligible for the study.

Enrolment was slated to close when about 100 evaluable study participants had been newly identified as HIV positive. Because of the estimated prevalence rates between 9.4% and 14.1% in Limpopo and Mupalanga Provinces respectively, and possible response rates under possible HIV+ persons, it was expected that the sample size would be under 2,000 based upon eligibility for inclusion.

### Tests and instructions

The OraQuick Rapid HIV-1/2 Antibody Test is a visually read, qualitative, in vitro lateral flow immunoassay for the detection of antibodies to HIV-1 and HIV-2. The device is currently approved for use with oral fluid specimens, whole blood specimens—collected either by venipuncture or fingerstick phlebotomy, and plasma specimens. The OraQuick HIV Self-Test used in the study was the same product as the current commercial test—OraQuick Rapid HIV-1/2 Antibody Test- with an additional users’ pamphlet. The OraQuick Rapid HIV-1/2 Antibody Test and the OraQuick HIV Self-Test were both from lot HIVCO-5244, expiry 28Feb2018. Instructions were provided in English, Afrikaans, Sepedi, Tsonga, Tswana, and Zulu languages together with explanatory pictures. After a month of enrolment the instructions were adapted due to frequency of similar procedural errors such as waiting time for reading the test result.

In the event of an HIV reactive OraQuick test, HIV status was confirmed using the standard rapid diagnostic testing algorithm in use in the testing centers by using the ABON HIV 1/2/O Tri-Line Human Immunodeficiency Virus Rapid Test.

### Health literacy

A health literacy score was assessed by four questions taken from two validated screening instruments, the Brief Estimate of Health Knowledge and Action (BEHKA)—HIV Version [18] and from the Set of Brief Screening Questions (SBSQ) [19, 20]. To keep the screening for health literacy as simple as possible and relevant for the local situation two questions dealing with written health information (questions 12 and 16) were taken from the SBSQ. Also two questions were selected from the BEHKA-HIV dealing with knowledge about CD4 cells as it was expected that people knew the meaning of a CD4-count (For list of questions and ratings see S1 File).

### Analysis

The primary endpoint was to establish the performance of the OraQuick HIV Self-Test in the hands of an untrained user. The primary efficacy endpoints were the binomial proportions for concordance of the study participants’ interpretation of their Self-Test results with the blinded trained user test results. Sensitivity is the proportion the blinded trained user test preliminary positive results read as positive by the study participants’ Self-Test. Specificity is the proportion the blinded trained user negative test results read as negative by the study participants’ Self-Test.

Secondary efficacy endpoints were the sensitivity and specificity among various subgroups.

Additional endpoints were the result with ABON HIV 1/2/O Tri-Line Human Immunodeficiency Virus Rapid Test, the trained user’s interpretation of the participant self-test result, and observational ratings of participant self-test performance. All difficulties and procedural errors were summed up to become a total score of procedural errors. A multiple linear regression analysis was performed to study the association of personal characteristics with procedural errors. (For list of questions see S1 File)

**Fig 1:**
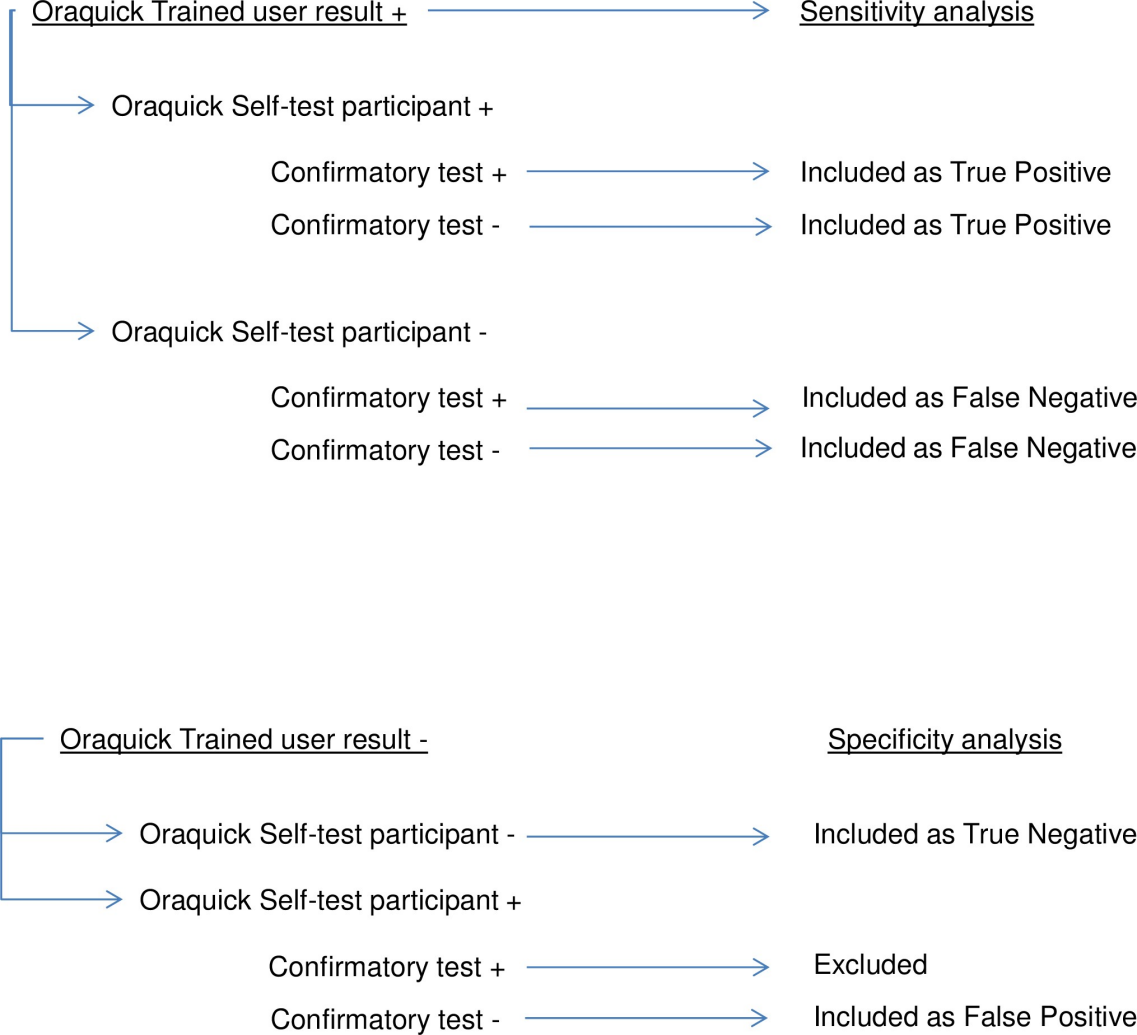
Flowchart sensitivity and specificity analysis.

The blinded trained user’s test result was the basis for determination of eligibility for inclusion of the study participant’s data in either the sensitivity or the specificity analysis. The sensitivity analysis included study participants who had positive blinded trained user results as reference test. The specificity analysis included study participants who had negative blinded trained user results. Study participants who had a positive oral fluid Self-Test (confirmed by second rapid finger pick test) and a negative blinded trained user test result would be excluded, but none had to be excluded for this reason. Study participants who had a positive Self-Test (indicating the need for confirmatory testing) and a negative trained user test result which was not confirmed would be included as false positives.(Fig 1)

All analyses were performed using IBM SPSS Version 22.

### Ethical statement

Ethical approval was obtained from the Health Sciences Ethics Review Committee, University of Pretoria under protocol number 394/2015. All participants opting for HIVST provided written (or witnessed thumbprint) informed consent as approved by the Ethics Review Committee.

## Results

### Participants

A total of 1,391 eligible study participants were included in the study, after exclusion of 18 non-eligible persons (9 younger than 18 or older than 50, 8 with a visit date last HIV-test being less than 6 months, 1 duplicate participant) and one other protocol deviation (second rapid test performed in preliminary negative HIV screening tests). Mean age was 27.9 (SD 8.05) years and they belonged primarily to the Pedi (North-Sotho) (600/1,390, 43.2%). Two thirds were female. A large majority of the study participants completed some form of education with 644/1,391 (46.3%) completing some high school and 478/1,391 (34.4%) completing high school. The months from last HIV-test ranged from 6 to 312 (or 26 years) for the population with a median of 13 months. Nine % (132/1,391) were never tested before. Most study participants were deemed adequately literate (score 7 to 10 on a scale of 10) (960/ 1,391, 69%). Their demographics are presented in Table 1.

**Table 1:** Characteristics of study participants.

	Overall	HIV-positive	HIV-negative
	N = 1,391	N = 113	N = 1,278
Age (mean, SD)	27.9 (8.1)	32.5 (8.1)	27.5 (7.9)
Gender (%)			
• Female	932 (67.0%)	93 (82.3%)	839 (65.6%)
• Male	459 (33.0%)	20 (17.7%)	439 (34.4%)
Education			
• Grade 1–8	142 (10.2%)	18 (15.9%)	124 (9.7%)
• Grade 9–11	644 (46.3%)	51 (45.1%)	593 (46.4%)
• Completed grade 12	478 (34.4%)	37 (32.7%)	441 (34.5%)
• College/University	127 (9.1%)	7 (6.2%)	120 (9.4%)
Health Literacy (mean, SD) (score min -2 –max 10)	7.37 (2.20)	7.55 (2.32)	7.36 (2.19)
Languages			
- Pedi	599 (43.1%)	40 (35.4%)	559 (43.7%)
- Sotho	200 (14.4%)	13 (11.5%)	187 (14.6%)
- Ndbele	168 (12.1%)	13 (11.5%)	155 (12.1%)
- Zulu	167 (12.0%)	24 (21.2%)	143 (11.2%)
- Tswana	102 (7.3%)	8 (7.1%)	94 (7.4%)
- Other	154 (11.1%)	15 (13.3%)	139 (10.9%)
Last HIV-test (months) (mean, SD)	20.31 (23.97)	31.04 (42.48)	19.31 (21.24)
(median, min-max)	13 (6–312)	14 (6–312)	13 (6–247)

Of the 1,391 eligible study participants, 113 (8.1%) had a positive OraQuick Professional test result read by the blinded trained user, 1,278 had a negative test result. All 113 positive Oraquick test results were confirmed reactive with the Abon finger prick test. Fifty-five from the 65 followed-up by telephone attended a health care provider for confirmation and treatment.

### Accuracy of oral Self-Test reading versus Trained user oral test

**Fig 2:**
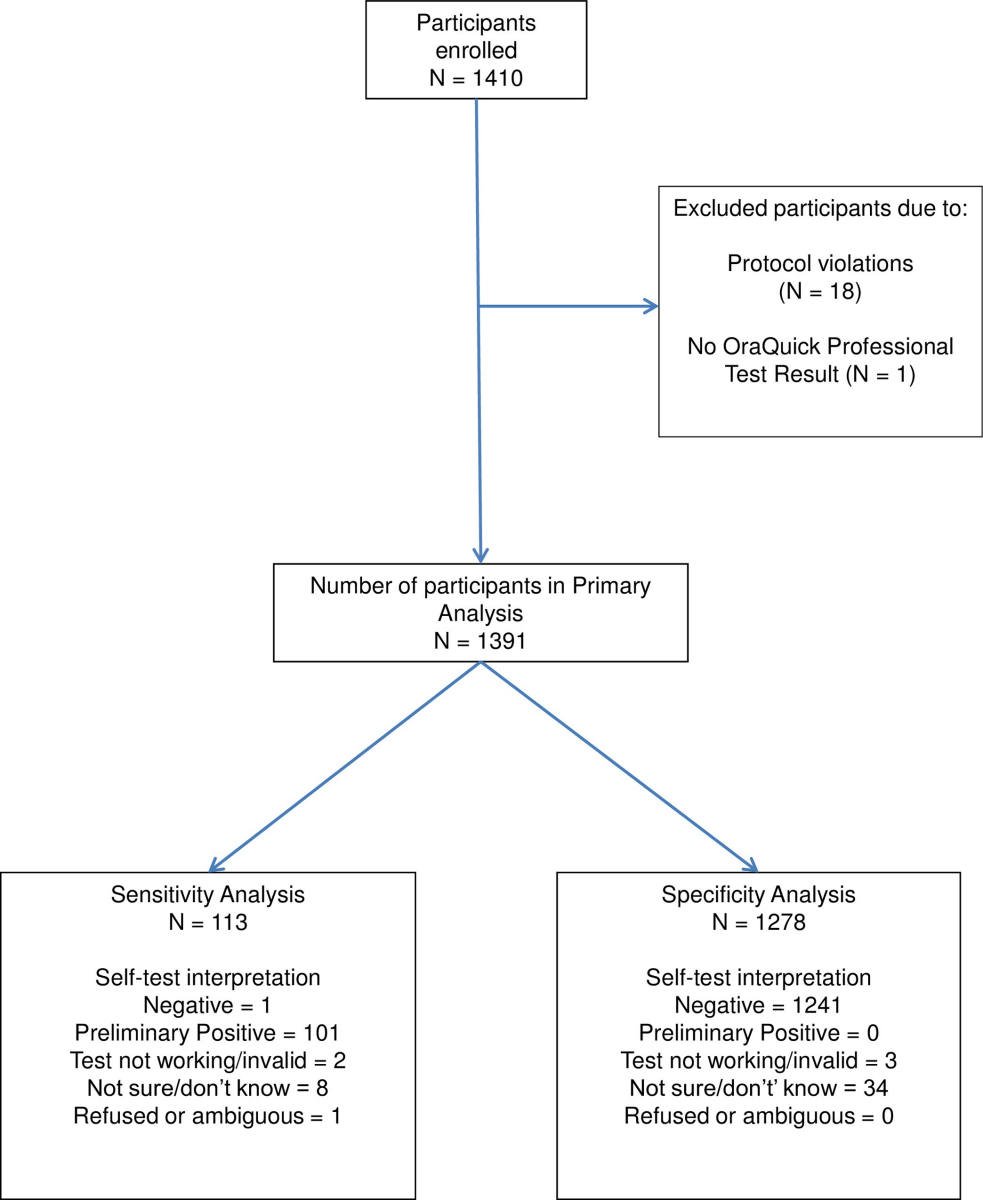
Study participant disposition.

From the 113 HIV-positive participants, 102 were included in the sensitivity analysis and from the 1,278 HIV-negative 1,241 were included in the specificity analysis. 48 study participants were not included in both analyses due to a Self-Test result read by the participant as invalid (n = 5), not sure/don’t know (n = 42), or refused/ambiguous (n = 1) (see Fig 2).

The sensitivity—101 in 102 (99.02%; 95%CI = 93.88–99.95%)—and specificity– 1,241 in 1,241 (100.0%; 95%CI = 99.62–100.0%)—of the OraQuick HIV Self-Test in untrained users are summarized in Table 2.

**Table 2:** Validity Oraquick Self-Test result versus professional user test result.

	Oraquick blinded professional test result	
	Preliminary positive	Negative
Participant Self-Test result:		
- Preliminary positive	101	0
- Negative	1	1,241
Validity measures	Sensitivity	Specificity
	99.02% (95% CI 93.88–99.95)	100% (95% CI 99.62–100)

### Feasibility

The 48 excluded Self-Tests in the accuracy analysis resulted in a test system failure rate of 3.45% (95% CI 2.56%-4.55%) in Self-Test participants. Two of the five test results read as invalid were overread by the observer as positive, three were indeed invalid. There was a higher percentage of participants that reported “not sure/don’t know” in the sensitivity analysis group than in the specificity analysis group, 7.1% (8/113) and 2.7% (34/1,278) respectively. Thirty-three were overread as negative, 8 as positive. Interrater agreement was 96.76% (kappa 0.82; 95% CI 0.77–0.87). The participants with test system failures were somewhat older than those without (mean 31.9, SD 9.9 vs 27.8, SD 7.9) and had a lower health literacy score (mean 7.16, SD 2.10 vs 7.38, SD 2.20). About half of them were anxious or distressed (22/48, 45.8% vs 324/1,391, 23.3%) and had at all levels more procedural difficulties and errors with the exception of swabbing.

Of the 1,391 subjects, 1,193 (84.6%) had at least one observation of difficulty or error with one or more of the test steps. Problems with self-execution of the test were mainly difficulties with not collecting the oral fluid sample properly (485/1,391, 34.9%), touching of the flat pad (396/1,391, 28.5%), and placing the tube in the plastic holder (389/1,391, 28.0%). (Table 3)

**Table 3:** Difficulties and errors observed during the self-test process.

Observation	Overall	
	N = 1,391	
	Yes	No
Was the participant able to find the test stick packet?	**99.80%**	0.20%
Did the participant remove test stick?	**99.60%**	0.30%
Did the participant remove the test tube?	**99.10%**	0.90%
Did the participant remove the cap from the tube?	**99.10%**	0.90%
Was the participant able to find test tube packet?	**98.70%**	1.30%
Did the participant read the information sheet?	**96.70%**	3.30%
Did the participant place stick in tube correctly?	**90.20%**	9.80%
Was it difficult to remove contents?	5.80%	**94.20%**
Did the participant have difficulties with the tube?	20.60%	**79.40%**
Did the participant place the tube in the holder?	**72.00%**	28.00%
Did the participant touch the flat pad?	28.50%	**71.40%**
Did the participate collect the oral fluid sample correctly?	**65.10%**	34.90%

Bold printed results are correct outcomes

While errors or difficulties in Self-Test execution by the untrained users were observed, only 4 tests did not provide a valid result as determined by the trained user’s interpretation of the Self-Test.

Initially it was observed that study participants had a tendency to read the Self-Test early instead of following the directions for use for timing between 20 and 40 minutes. Once the directions for use were updated to simplify the time symbolism and place it earlier in the sequence of test steps (to allow for planning by the study participant), adherence to time improved (improvement for 20 to 40 minutes was +32.1%, for 18 to 20 minutes was +20.5%, with under 15 minutes dropping by 50.5%). (Table 4)

**Table 4:** Reading time self-test according 2 versions of instructions.

Reading Time (in minutes)	Overall N = 1,391	1^st^ Instructions N = 356	2^nd^ Instructions N = 1,035
**<15**	224 (16.1%)	192 (53.9%)	32 (3.1%)
**15 to <18**	208 (15.0%)	59 (16.6%)	149 (14.5%)
**18 to <20**	400 (28.7%)	48 (13.5%)	352 (33.9%)
**20 to 40**	559 (40.2%)	57 (16.0%)	502 (48.5%)
**> 40**	0 (0.0%)	0 (0.0%)	0 (0.0%)

Age, education and health literacy were independently associated with the sum score of procedural errors and difficulties. Health literacy was in all analyses the strongest independent factor, also when analysing difficulties and procedural errors separately. (Table 5)

**Table 5:** Multivariate linear regression models for demographic factors related with processing difficulties and errors scores.

Factor	Processing difficulties^a^		Processing errors^b^		Total processing difficulties and errors^c^	
	Regression coefficient (SD)	p-value	Regression coefficient (SD)	p-value	Regression coefficient (SD)	p-value
Constant	0.588 (0.091)		2.526 (0.215)		2.653 (0.225)	
Health literacy	-0.028 (0.007)	< 0.001	-0.120 (0.017)	<0.001	-0.121 (0.018)	<0.001
Education	-0.017 (0.007)	0.020	-0.104 (0.017)	<0.001	-0.100 (0.018)	<0.001
Age	0.006 (0.002)	0.002	0.021 (0.004)	<0.001	0.022 (0.005)	<0.001
Female			0.155 (0.075)	0.038		

adjusted R^2^

a: 0.026

b: 0.101

c: 0.093

#### Acceptability

Of the 1,278 HIV-negative participants who answered a testing intent question series, 768 (60.1%) answered that they most likely or definitely intend to seek clinic testing, however 1,226 (96%) answered that they would most likely or definitely use a Self-Test if it becomes available. Additionally, 1,221 (95.5%) would most likely or definitely recommend a Self-Test to sexual partners.

## Discussion

The primary endpoint was to establish the performance of the OraQuick HIV Self-Test using oral fluid in the hands of an untrained user in a rural setting in a population with a relatively lower educational level. Sensitivity (99.02%) and specificity (100.0%) were high. Validity figures were similar to the ones in a supervised oral Self-Test study in rural Kwazulu-Natal, whereby the Self-Test was demonstrated to the participants by counsellors, with a sensitivity of 98.7% and specificity of 100% [15, 21]. Studies reporting the validity of the OraQuick Rapid HIV-1/2 Antibody Test in Zambia and Malawi reported respectively a sensitivity of 98.7 and 93.6% and a specificity of 99.8 and 99.9% [22, 23].

Interrater-agreement in our study was lower than in the rural study where the Self-Test was demonstrated: 96.8% (kappa 0.82) versus 99.8% (kappa 0.99) [15]. Reading and interpreting the test result correctly resulted in a failure rate of only 3.5%. The percentage that was not sure or did not know how to interpret the test result was significantly higher among HIV-positive participants. Maybe they were in denial and therefore did not want to interpret their results. It shows that not only users who read their positive Self-Test result correctly will need access to post-test counselling services.

In a usability study comparing 5 different Self-Tests (1 oral) performed in Kenia, Malawi and South Africa, 39% conducted all steps correctly with the oral Self-Test compared to 15% in our study, whereby we included the difficulties they had to follow the procedures. But in our study 65% collected the oral fluid sample correctly compared to 39% in the other study [12]. In another study in urban setting in Malawi using an oral Self-Test after a short demonstration, 10% requested help and 10% made errors [14]. The study in Kwazulu-Natal defined a user error rate only when a test had to be repeated (0.09%) [15]. While still under observation, we tried to come closer to the circumstances a Self-Test will be performed without any additional explanation or demonstration. The still high percentage of difficulties with the device–especially with putting the tube with the liquid in a plastic holder–reflects the need for more research on device usability. As only 4 in 1,391 Self-Tests were eventually invalid as over-read by the trained user, the Self-Test seems to be robust against any procedural error observed. Although procedural errors seem not to affect the validity of the Self-Test compared with the test outcome by a professional user, instruction leaflets may need still more attention especially regarding pictures and symbols used. This was clearly demonstrated by the significant improvement in the reading time respected before reading the test result. In the study of Peck et al. 69% did not wait for the required time compared to 51.5% in our study after adaptation of the instructions [12]. As lower health literacy, lower education and higher age are associated with procedural errors, producers of devices should keep their Self-Tests as feasible as possible with simple steps and easy procedures and develop very simple accompanying instructions [12]. Interpretation of pictures should not be taken for granted or generalizable in all populations.

As a Self-Test should be seen and sold as a screening test, one may expect that users with a positive test result will seek for confirmation of their Self-Test outcome as long as they are ‘ready’ for it [21]. The outcome of this study shows that their result has a high chance to be confirmed, taking into consideration the sensitivity of the OraQuick HIV Self-Test in other studies [15].

The linkage to care was confirmed in 55 HIV-positive participants, but only 65 could be contacted by phone. Also in an actual running Home Based Testing programme in the same area it remains difficult to monitor linkage to care with registered participants by phone only. In the programme teams of two counsellors are visiting households in the local communities and invite family members to be tested voluntarily with the oral test. Often referred HIV-positive participants can only be reached by voicemail or they do not want to answer the call. The programme uses re-visits at home and visits at referral health centers to get additional information after informed consent. The more difficult it will be to assess linkage to care when people are using self-tests in the privacy of their homes.

### Strengths and limitations

Although the Self-Test was delivered to the participants without any introduction, the presence of an observer monitoring the process might have made participants uncomfortable and nervous. This might have led to a higher number of procedural errors as about half (45%) of participants with system failures were distressed or anxious versus 22% in the others. Conversely, the presence of the other person in the room may explain better results, whereby participants may have been more careful knowing someone was in the room or going to read their results again, although the amount of procedural errors suggests the contrary. The reality of self-testing in an own familiar and private environment may result in less errors. On the other hand participants were invited to outreach places in their various communities and are likely to represent those of the local rural community that are eager to be tested and know their status and might be future buyers of self-tests once they become available and affordable.

## Conclusion

The OraQuick HIV Self-Test seems to be robust and provide reliable results in a rural population. Self-testing remains prone to procedural errors and difficulties requiring a robust test. Self-Test instructions and procedures require simple steps, easy devices and simple and clear pictural instructions to be tested in various target populations. Still the performance of self-testing in private circumstances in local African populations might need more evidence.

## Supporting information

S1 File Supplemental data. (DOCX)

S1 Dataset Minimum dataset. (DOCX)
