# Supplementary material for: Feasibility and robustness of an oral HIV self-test in a rural community in South-Africa: An observational diagnostic study
Source: PLoS One. 2019 Apr 15;14(4):e0215353. doi: 10.1371/journal.pone.0215353 (PMC6464222; doi:10.1371/journal.pone.0215353)
Supplement: S1 File — (DOCX) [file pone.0215353.s001.docx]

**Supplemental data**

**Health literacy**

Health literacy level was assessed by asking the following questions:

How often do you have problems learning about your medical condition because of difficulty understanding written information?

1 2 3 4

Always Never

How often do you have someone (like family member, friend or caregiver) help you read hospital materials?

1 2 3 4

Always Never

Did you ever hear about a CD4 count? Do you know what it means?

1 -1 0

Answer: Correct Not correct Do not know

If a HIV-patient is treated is the CD4 count expected to go up or down?

1 -1 0

Answer: Up Down Do not know

These answers were scored and the total score was assigned a rating of:

- Inadequate (score of -2 to 2)
- Marginal (score of 3 to 6)
- Adequate (score of 7 to 10)

**Observational ratings of study participant-Self-Test performance**

1. Did the study participant read the information sheet? No=1 / Yes = 0
2. Was it difficult for the study participant to remove the inner box? No=0 / Yes = 1
3. Was the study participant able to find the test tube packet? No=1 / Yes = 0
4. Did the study participant remove the test tube from the packet? No=1 / Yes = 0
5. Did the study participant remove the cap from the test tube? No=1 / Yes = 0
6. Did the study participant place the test tube in the holder? No=1 / Yes = 0
7. Did the study participant have any difficulty with the test tube? (specify if “yes”) No=0 / Yes = 1
8. Was the study participant able to find the test stick packet? No=1 / Yes = 0
9. Did the study participant remove the test stick from the packet? No=1 / Yes = 0
10. Did the study participant touch the flat pad? No=0 / Yes = 1
11. Did the study participant collect the sample correctly (1x upper and lower swab)? (If no, specify) No=1 / Yes = 0
12. Did the study participant place the test stick in the test tube correctly? (If not, specify) No=1 / Yes = 0

Score of procedural errors = sum of questions 1 to 12
