# Supplementary material for: Feasibility and robustness of an oral HIV self-test in a rural community in South-Africa: An observational diagnostic study
Source: PLoS One. 2019 Apr 15;14(4):e0215353. doi: 10.1371/journal.pone.0215353 (PMC6464222; doi:10.1371/journal.pone.0215353)
Supplement: S1 Dataset — (DOCX) [file pone.0215353.s002.docx]

**Minimum dataset**

Summary of Self-Test Distribution

| **Self-test interpretation participant * Test result parallel test Crosstabulation** | | | | | |
| --- | --- | --- | --- | --- | --- |
|  | | | **Testresult parallel test** | | **Total** |
|  |  |  | **Negative** | **Positive** |  |
| **Self-test interpretation participant** | **Negative** | **Count** | **1241** | **1** | **1242** |
|  |  | **% within Testresult parallel test** | **97.1%** | **0.9%** | **89.3%** |
|  | **Preliminary positive** | **Count** | **0** | **101** | **101** |
|  |  | **% within Testresult parallel test** | **0.0%** | **89.4%** | **7.3%** |
|  | **Test not workin / invalid** | **Count** | **3** | **2** | **5** |
|  |  | **% within Testresult parallel test** | **0.2%** | **1.8%** | **0.4%** |
|  | **Not sure / dont know** | **Count** | **34** | **8** | **42** |
|  |  | **% within Testresult parallel test** | **2.7%** | **7.1%** | **3.0%** |
|  | **Refused / ambiguous** | **Count** | **0** | **1** | **1** |
|  |  | **% within Testresult parallel test** | **0.0%** | **0.9%** | **0.1%** |
| **Total** | | **Count** | **1278** | **113** | **1391** |
|  |  | **% within Testresult parallel test** | **100.0%** | **100.0%** | **100.0%** |

Test System Failures

| **Self-test interpretation participant * Self Test result Crosstabulation** | | | | | | |
| --- | --- | --- | --- | --- | --- | --- |
|  | | | **Selftest result** | | | **Total** |
|  |  |  | **Negative** | **Positive** | **Invalid** |  |
| **Self-test interpretation participant** | **Negative** | **Count** | **1240** | **1** | **1** | **1242** |
|  |  | **% within Self-test interpretation participant** | **99.8%** | **0.1%** | **0.1%** | **100.0%** |
|  |  | **% within Selftest result** | **97.3%** | **0.9%** | **25.0%** | **89.3%** |
|  | **Preliminary positive** | **Count** | **0** | **101** | **0** | **101** |
|  |  | **% within Self-test interpretation participant** | **0.0%** | **100.0%** | **0.0%** | **100.0%** |
|  |  | **% within Selftest result** | **0.0%** | **89.4%** | **0.0%** | **7.3%** |
|  | **Test not working / invalid** | **Count** | **1** | **2** | **2** | **5** |
|  |  | **% within Self-test interpretation participant** | **20.0%** | **40.0%** | **40.0%** | **100.0%** |
|  |  | **% within Selftest result** | **0.1%** | **1.8%** | **50.0%** | **0.4%** |
|  | **Not sure / dont know** | **Count** | **33** | **8** | **1** | **42** |
|  |  | **% within Self-test interpretation participant** | **78.6%** | **19.0%** | **2.4%** | **100.0%** |
|  |  | **% within Selftest result** | **2.6%** | **7.1%** | **25.0%** | **3.0%** |
|  | **Refused / ambiguous** | **Count** | **0** | **1** | **0** | **1** |
|  |  | **% within Self-test interpretation participant** | **0.0%** | **100.0%** | **0.0%** | **100.0%** |
|  |  | **% within Selftest result** | **0.0%** | **0.9%** | **0.0%** | **0.1%** |
| **Total** | | **Count** | **1274** | **113** | **4** | **1391** |
|  |  | **% within Self-test interpretation participant** | **91.6%** | **8.1%** | **0.3%** | **100.0%** |
|  |  | **% within Selftest result** | **100.0%** | **100.0%** | **100.0%** | **100.0%** |

Trained User Self-Test and OraQuick Professional

| **Self Test result** | | | | | |
| --- | --- | --- | --- | --- | --- |
|  | | **Frequency** | **Percent** | **Valid Percent** | **Cumulative Percent** |
| **Valid** | **Negative** | **1274** | **91.6** | **91.6** | **91.6** |
|  | **Positive** | **113** | **8.1** | **8.1** | **99.7** |
|  | **Invalid** | **4** | **.3** | **.3** | **100.0** |
|  | **Total** | **1391** | **100.0** | **100.0** |  |
| **Test result parallel test** | | | | | |
|  | | **Frequency** | **Percent** | **Valid Percent** | **Cumulative Percent** |
| **Valid** | **Negative** | **1278** | **91.9** | **91.9** | **91.9** |
|  | **Positive** | **113** | **8.1** | **8.1** | **100.0** |
|  | **Total** | **1391** | **100.0** | **100.0** |  |

Intention Tables

| Likely get tested again | | | | | |
| --- | --- | --- | --- | --- | --- |
|  | | Frequency | Percent | Valid Percent | Cumulative Percent |
| Valid | Not at all | 260 | 18.7 | 20.2 | 20.2 |
|  | Likely | 258 | 18.5 | 20.0 | 40.2 |
|  | Most likely | 307 | 22.1 | 23.8 | 64.1 |
|  | Definitely | 463 | 33.3 | 35.9 | 100.0 |
|  | Total | 1288 | 92.6 | 100.0 |  |
| Missing | System | 103 | 7.4 |  |  |
| Total | | 1391 | 100.0 |  |  |

| Likely to test again with home test | | | | | |
| --- | --- | --- | --- | --- | --- |
|  | | Frequency | Percent | Valid Percent | Cumulative Percent |
| Valid | Not at all | 18 | 1.3 | 1.4 | 1.4 |
|  | Likely | 37 | 2.7 | 2.9 | 4.3 |
|  | Most likely | 220 | 15.8 | 17.1 | 21.4 |
|  | Definitely | 1013 | 72.8 | 78.6 | 100.0 |
|  | Total | 1288 | 92.6 | 100.0 |  |
| Missing | System | 103 | 7.4 |  |  |
| Total | | 1391 | 100.0 |  |  |
| Likely to use home test sexual partner | | | | | |
|  | | Frequency | Percent | Valid Percent | Cumulative Percent |
| Valid | Not at all | 15 | 1.1 | 1.2 | 1.2 |
|  | Likely | 44 | 3.2 | 3.4 | 4.6 |
|  | Most likely | 197 | 14.2 | 15.3 | 19.9 |
|  | Definitely | 1032 | 74.2 | 80.1 | 100.0 |
|  | Total | 1288 | 92.6 | 100.0 |  |
| Missing | System | 103 | 7.4 |  |  |
| Total | | 1391 | 100.0 |  |  |

**Difficulty Seeing With Vision Correction**

| **Seeing** | | | | | |
| --- | --- | --- | --- | --- | --- |
|  | | **Frequency** | **Percent** | **Valid Percent** | **Cumulative Percent** |
| **Valid** | **No difficulty** | **1096** | **78.8** | **79.2** | **79.2** |
|  | **Some difficulty** | **263** | **18.9** | **19.0** | **98.3** |
|  | **A lot of difficulty** | **24** | **1.7** | **1.7** | **100.0** |
|  | **Total** | **1383** | **99.4** | **100.0** |  |
| **Missing** | **System** | **8** | **.6** |  |  |
| **Total** | | **1391** | **100.0** |  |  |

**Characteristics of SUB-GROUPS for Sensitivity and Specificity Analysis**

| **Summary of Age** | | | | | |
| --- | --- | --- | --- | --- | --- |
| **AGE** | **N** | **Minimum** | **Maximum** | **Mean** | **Std. Deviation** |
| **Specificity** | **1278** | **18.00** | **49.00** | **27.5133** | **7.92700** |
| **Sensitivity** | **113** | **18.00** | **49.00** | **32.4867** | **8.09113** |

| **Summary of Months Since Last HIV Test** | | | | | | | | | | |
| --- | --- | --- | --- | --- | --- | --- | --- | --- | --- | --- |
| **MONTHS SINCE LAST HIV TEST** | | | **N** | **Minimum** | | **Maximum** | | | **Mean** | **Std. Deviation** |
| **Specificity** | | | **1152** | **6.00** | | **247.00** | | | **19.3177** | **21.24839** |
| **Sensitivity** | | | **106** | **6.00** | | **312.00** | | | **31.0472** | **42.48101** |
| **Summary of Gender** | | | | | | | |  |  |  |
| **GENDER** | | **Frequency** | | | **Percent** | |  |  |  |  |
| **Specificity** | **Male** | **439** | | | **34.4** | |  |  |  |  |
|  | **Female** | **839** | | | **65.6** | |  |  |  |  |
|  | **Total** | **1278** | | | **100.0** | |  |  |  |  |
| **Sensitivity** | **Male** | **20** | | | **17.7** | |  |  |  |  |
|  | **Female** | **93** | | | **82.3** | |  |  |  |  |
|  | **Total** | **113** | | | **100.0** | |  |  |  |  |

| **Summary of Education** | | | | |
| --- | --- | --- | --- | --- |
| **EDUCATION** | | **Frequency** | **Percent** |  |
| Specificity | **Primary** | **124** | **9.7** |  |
|  | **High School** | **593** | **46.4** |  |
|  | **Completed Matric** | **441** | **34.5** |  |
|  | **Completed College or University** | **120** | **9.4** |  |
|  | **Total** | **1278** | **100.0** |  |
| Sensitivity | **Primary** | **18** | **15.9** |  |
|  | **High School** | **51** | **45.1** |  |
|  | **Completed Matric** | **37** | **32.7** |  |
|  | **Completed College or University** | **7** | **6.2** |  |
|  | **Total** | **113** | **100.0** |  |

| **Summary of Language** | | | | |
| --- | --- | --- | --- | --- |
| **LANGUAGE** | | **Frequency** | **Percent** |  |
| **Specificity** | **Missing** | **1** | **.1** |  |
|  | **Afrikaans** | **1** | **.1** |  |
|  | **Ndebele** | **155** | **12.1** |  |
|  | **Other** | **20** | **1.6** |  |
|  | **Pedi** | **559** | **43.7** |  |
|  | **Sotho** | **187** | **14.6** |  |
|  | **Tsonga** | **52** | **4.1** |  |
|  | **Tswana** | **94** | **7.4** |  |
|  | **Tswati** | **41** | **3.2** |  |
|  | **Venda** | **11** | **.9** |  |
|  | **Xhosa** | **14** | **1.1** |  |
|  | **Zulu** | **143** | **11.2** |  |
|  | **Total** | **1278** | **100.0** |  |
| **Sensitivity** | **Ndebele** | **13** | **11.5** |  |
|  | **Other** | **1** | **.9** |  |
|  | **Pedi** | **40** | **35.4** |  |
|  | **Sotho** | **13** | **11.5** |  |
|  | **Tsonga** | **7** | **6.2** |  |
|  | **Tswana** | **8** | **7.1** |  |
|  | **Tswati** | **6** | **5.3** |  |
|  | **Xhosa** | **1** | **.9** |  |
|  | **Zulu** | **24** | **21.2** |  |
|  | **Total** | **113** | **100.0** |  |

| **Summary of Health Literacy** | | | | |
| --- | --- | --- | --- | --- |
| **HEALTH LITERACY** | | **Frequency** | **Percent** |  |
| **Specificity** | **1.00** | **3** | **.2** |  |
|  | **2.00** | **12** | **.9** |  |
|  | **3.00** | **20** | **1.6** |  |
|  | **4.00** | **65** | **5.1** |  |
|  | **5.00** | **73** | **5.7** |  |
|  | **6.00** | **153** | **12.0** |  |
|  | **7.00** | **105** | **8.2** |  |
|  | **8.00** | **431** | **33.7** |  |
|  | **9.00** | **71** | **5.6** |  |
|  | **10.00** | **275** | **21.5** |  |
|  | **Total** | **1208** | **94.5** |  |
| **Sensitivity** | **2.00** | **2** | **1.8** |  |
|  | **3.00** | **1** | **.9** |  |
|  | **4.00** | **5** | **4.4** |  |
|  | **5.00** | **3** | **2.7** |  |
|  | **6.00** | **15** | **13.3** |  |
|  | **7.00** | **5** | **4.4** |  |
|  | **8.00** | **32** | **28.3** |  |
|  | **9.00** | **9** | **8.0** |  |
|  | **10.00** | **32** | **28.3** |  |
|  | **Total** | **104** | **92.0** |  |

| **Summary of Ability to See** | | | | |
| --- | --- | --- | --- | --- |
| **DIFFICULTY SEEING** | | **Frequency** | **Percent** |  |
| **Specificity** | **No difficulty** | **1019** | **79.7** |  |
|  | **Some difficulty** | **233** | **18.2** |  |
|  | **A lot of difficulty** | **18** | **1.4** |  |
|  | **Total** | **1270** | **99.4** |  |
| **Sensitivity** | **No difficulty** | **77** | **68.1** |  |
|  | **Some difficulty** | **30** | **26.5** |  |
|  | **A lot of difficulty** | **6** | **5.3** |  |
|  | **Total** | **113** | **100.0** |  |

| **Summary of Glasses Use** | | | |
| --- | --- | --- | --- |
| **GLASSES** | | **Frequency** | **Percent** |
| **Specificity** | **No** | **1220** | **95.5** |
|  | **Yes** | **58** | **4.5** |
|  | **Total** | **1278** | **100.0** |
| **Sensitivity** | **No** | **104** | **92.0** |
|  | **Yes** | **9** | **8.0** |
|  | **Total** | **113** | **100.0** |

| **Summary of Ocular Health** | | | |
| --- | --- | --- | --- |
| **CONDITION THAT AFFECTS VISION** | | **Frequency** | **Percent** |
| **Specificity** | **No** | **1238** | **96.9** |
|  | **Yes** | **39** | **3.1** |
|  | **Total** | **1277** | **99.9** |
| **Sensitivity** | **No** | **109** | **96.5** |
|  | **Yes** | **4** | **3.5** |
|  | **Total** | **113** | **100.0** |
